# Prediction of prostate tumour hypoxia using pre-treatment MRI-derived radiomics: preliminary findings

**DOI:** 10.1007/s11547-023-01644-3

**Published:** 2023-05-17

**Authors:** Jim Zhong, Russell Frood, Alan McWilliam, Angela Davey, Jane Shortall, Martin Swinton, Oliver Hulson, Catharine M. West, David Buckley, Sarah Brown, Ananya Choudhury, Peter Hoskin, Ann Henry, Andrew Scarsbrook

**Affiliations:** 1 Leeds Institute of Medical Research, University of Leeds, Leeds, UK; 2 Department of Radiology, Leeds Cancer Centre, St James’s University Hospital, Leeds Teaching Hospitals National Health Service (NHS) Trust, Beckett Street, Leeds, LS9 7TF UK; 3 Division of Cancer Sciences, School of Medical Sciences, Faculty of Biology, Medicine and Health, The University of Manchester, Manchester, UK; 4 Department of Radiotherapy Related Research, The Christie National Health Service (NHS) Foundation Trust, Manchester, UK; 5 Biomedical Imaging, Leeds Institute of Cardiovascular and Metabolic Medicine, University of Leeds, Leeds, UK; 6 Leeds Cancer Research UK Clinical Trials Unit, Leeds Institute of Clinical Trials Research (LICTR), University of Leeds, Leeds, UK; 7 Department of Clinical Oncology, Leeds Cancer Centre, St James’s University Hospital, Leeds Teaching Hospitals National Health Service (NHS) Trust, Beckett Street, Leeds, LS9 7TF UK

**Keywords:** Prostate cancer, Hypoxia, MRI, Radiomics, Radiogenomics, Machine learning

## Abstract

**Purpose:**

To develop a machine learning (ML) model based on radiomic features (RF) extracted from whole prostate gland magnetic resonance imaging (MRI) for prediction of tumour hypoxia pre-radiotherapy.

**Material and methods:**

Consecutive patients with high-grade prostate cancer and pre-treatment MRI treated with radiotherapy between 01/12/2007 and 1/08/2013 at two cancer centres were included. Cancers were dichotomised as normoxic or hypoxic using a biopsy-based 32-gene hypoxia signature (Ragnum signature). Prostate segmentation was performed on axial T2-weighted (T2w) sequences using RayStation (v9.1). Histogram standardisation was applied prior to RF extraction. PyRadiomics (v3.0.1) was used to extract RFs for analysis. The cohort was split 80:20 into training and test sets. Six different ML classifiers for distinguishing hypoxia were trained and tuned using five different feature selection models and fivefold cross-validation with 20 repeats. The model with the highest mean validation area under the curve (AUC) receiver operating characteristic (ROC) curve was tested on the unseen set, and AUCs were compared via DeLong test with 95% confidence interval (CI).

**Results:**

195 patients were included with 97 (49.7%) having hypoxic tumours. The hypoxia prediction model with best performance was derived using ridge regression and had a test AUC of 0.69 (95% CI: 0.14). The test AUC for the clinical-only model was lower (0.57), but this was not statistically significant (*p* = 0.35). The five selected RFs included textural and wavelet-transformed features.

**Conclusion:**

Whole prostate MRI-radiomics has the potential to non-invasively predict tumour hypoxia prior to radiotherapy which may be helpful for individualised treatment optimisation.

**Supplementary Information:**

The online version contains supplementary material available at 10.1007/s11547-023-01644-3.

## Introduction

Prostate cancer is the commonest malignancy in men and a major cause of cancer-related death [1]. Radiation therapy (RT), including external beam radiation therapy (EBRT) and brachytherapy (BT), is an effective treatment for localised prostate cancer [2]. Despite advances in diagnostic imaging and RT delivery techniques, treatment failure remains common with biochemical failure occurring in almost half of high-risk patients at 10 years [3–5].

Tumour hypoxia, a low oxygen environment, is associated with RT resistance and metastatic disease in prostate cancer [6–9]. Identifying tumour hypoxia may help with patient selection for radiation boosting. Current methods of assessing hypoxia, such as using prostate biopsy samples to identify gene-based hypoxia biomarkers, or oxygen electrodes, are invasive and hindered by sampling errors due to multi-focal tumours and intra-tumoral heterogeneity [10]. Magnetic resonance imaging (MRI) offers a potential non-invasive method of assessing hypoxia that allows the whole prostate to be measured and assessed over time, i.e. before, during and following treatment to monitor response.

Radiomics is a quantitative method of imaging analysis using data-characterisation algorithms to derive imaging biomarkers [11]. Imaging-based radiogenomics offers promise in bridging the gap between medical imaging and histopathological or molecular/gene signatures, by integrating data generated from complementary data sources to improve the accuracy of predictive models [12]. Machine learning (ML) models based on radiomic features (RF) extracted from T2-weighted (T2w) prostate MRI have demonstrated good performance for detecting clinically significant cancer [13]. Radiomic signatures have also been shown to accurately predict molecular subtypes of cancers associated with a more invasive phenotype [14]. The application of ML to radiogenomic studies provides novel insights into tumour biology and has been evaluated in multiple tumour types [15–17]. Identifying hypoxia on T2w prostate MRI also has treatment implications given the role of MRI-guided RT, which already incorporates a T2w sequence into the standard workflow, therefore there is potential for dose escalation based on an imaging radiogenomic approach.

The aim of this study was to develop a ML model based on RFs extracted from whole gland prostate MRI for prediction of tumour hypoxia pre-radiotherapy.

## Materials and methods

### Dataset and study population

This retrospective study was approved by the United Kingdom North West Research Ethics Committee (Validation and qualification of a multiplex hypoxia biomarker for radiotherapy individualisation in prostate cancer study (IRAS 15/NW/0559)). Informed consent was obtained from all patients.

The study cohort consisted of 195 consecutive patients with histologically confirmed high-risk prostate cancer treated between 01/12/2007 and 31/08/2013 at either < Institution A > with EBRT (74 Grey (Gy) in 37 fractions) (*N* = 100) or at < Institution B > with EBRT (57 Gy in 19 fractions) or EBRT (37.5 Gy in 15 fractions) plus high dose rate (HDR) brachytherapy (BT) boost (single fraction 15 Gy) (*N* = 95).

Inclusion criteria were: (a) male patients with prostate cancer aged at least 18 years; (b) primary radiotherapy to treat their prostate cancer (either BT or EBRT); (c) available pre-treatment MRI and hypoxia gene signature data; (d) available clinical features (patient age, International Society of Urological Pathology (ISUP) grade, prostate specific antigen (PSA) and T-stage).

### MRI acquisition

All patients underwent prostate MRI on 1.5 T MRI scanners which included a minimum of an axial T2w sequence encompassing the whole prostate. Imaging was performed using multiple different MRI scanners. Specific scanner acquisition parameters are listed in Supplementary Material Table 1.

**Table 1:** Demographics of the training and test cohort

Characteristics	Training Cohort (*n* = 156)* N * (%)	Test Cohort (*n* = 39)* N* (%)	*p*-value
Age* (years)	69.9 (IQR = 8.4)	68.0 (IQR = 10.0)	0.48
PSA (ng/mL)*	19.5 (IQR = 18.5)	21.0 (IQR = 17.0)	0.45
*ISUP*			
1	4 (2.5)	1 (2.4)	0.69
2	60 (38.8)	15 (31.7)	
3	27 (20.6)	10 (12.2)	
4	17 (8.1)	10 (12.2)	
5	48 (30.0)	11 (39.0)	
*T-stage*			
T1	4 (2.6)	1 (2.6)	0.91
T2	30 (19.2)	9 (23.1)	
T3	121 (77.6)	29 (74.4)	
T4	1 (0.6)	0	
*Hypoxia*			
Yes	78 (50.0)	19 (48.7)	0.89
No	78 (50.0)	20 (51.3)	

PSA = Prostate Specific Antigen, ISUP = International Society of Urological Pathology, T-stage = Tumour stage

^*^Median (IQR = interquartile range)

### Hypoxia gene signature

All patients were grouped into normoxia and hypoxia groups based on their pre-treatment prostate biopsy which was used as the ground truth for hypoxia status. The ribonucleic acid (RNA) from formalin-fixed, paraffin-embedded prostate biopsy specimens was extracted, and samples were processed using Affymetrix GeneChip (Clariom S Array) to calculate the expression of a 32-gene prostate hypoxia signature, based on pimonidazole staining (Ragnum signature) [9]. The gene enrichment analysis and construction of the gene signature is described by Ragnum et al. [9]. The normoxia and hypoxia split was based on a previously validated threshold [18].

### Study pipeline

Adherence was made to the Checklist for Artificial Intelligence in Medical Imaging (CLAIM) (Supplementary Material), a tool for assessing the quality of multivariate prediction models involving ML techniques [19].

### Image segmentation

All imaging data were de-identified using a data masking method. The whole prostate gland and prostate tumour (if visible) were manually segmented by an experienced radiologist and confirmed by a specialist Uroradiologist. Segmentation was performed using RayStation (v9.1). Exported DICOM images were converted to Neuroimaging Informatics Technology Initiative (NIfTI) files and exported into PyRadiomics (v3.0.1) for analysis [20]. The Nyúl method, a histogram intensity-based normalisation technique, was applied to MRI data to render the dynamic signal intensity ranges comparable prior to RF extraction [21, 22].

**Fig. 1:**
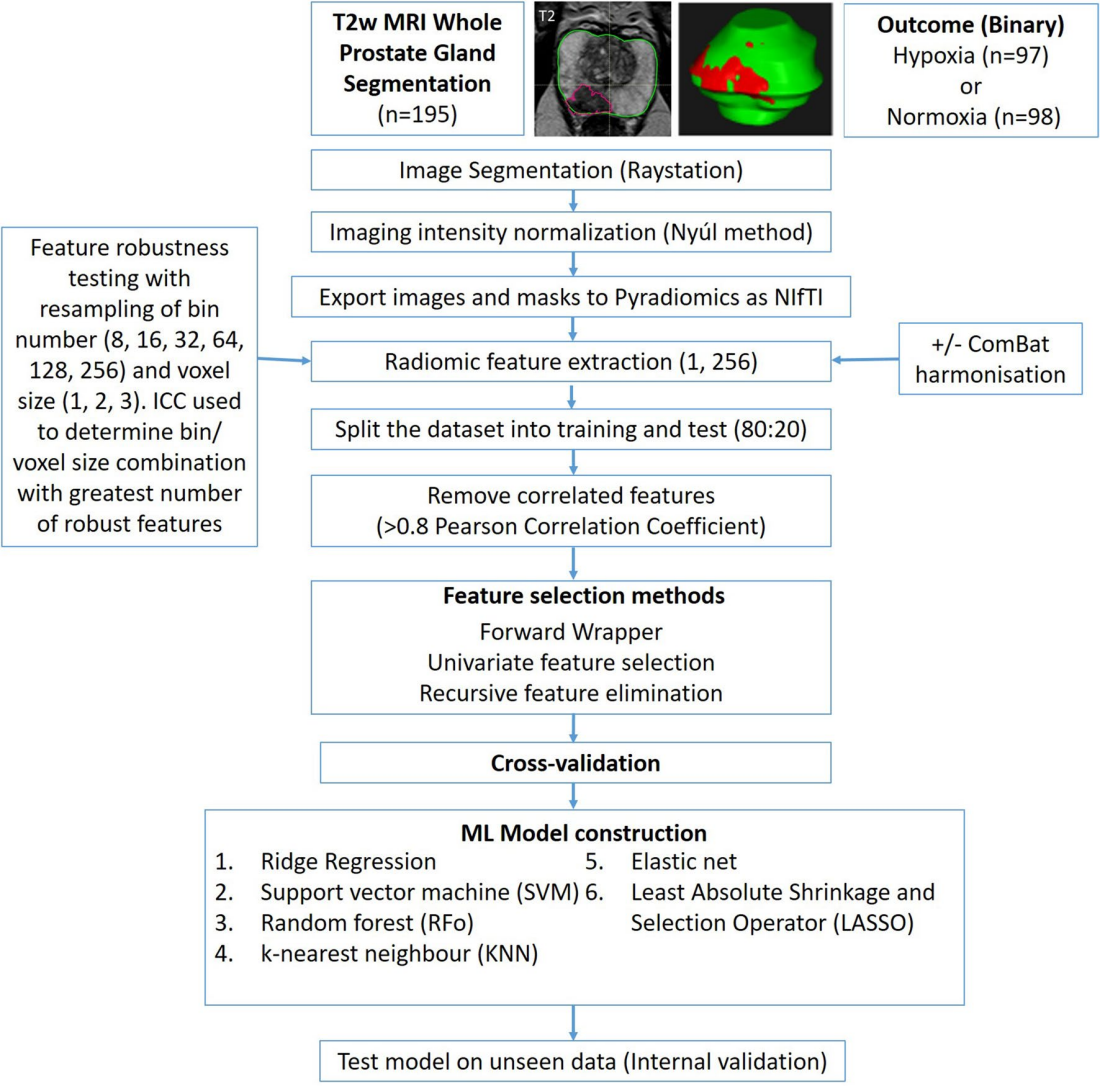
Flowchart demonstrating the methodological pipeline for the T2w MRI whole prostate gland radiomic model for predicting hypoxia Legend: NIfTI = Neuroimaging Informatics Technology Initiative, ICC = intraclass correlation coefficient, ComBat = imaging harmonisation method, LASSO = Least Absolute Shrinkage and Selection Operator, RFo = Random Forest.

A flowchart illustrating the methodological pipeline for RF-derived hypoxia prediction from segmentation through to ML model construction is shown in Fig. 1.

### Feature extraction

Eight RF classes [20] were extracted from each segmented region of interest (ROI) using PyRadiomics (v3.0.1) (https://pyradiomics.readthedocs.io/en/latest/index.html, accessed 09/02/2023). All RFs extracted and filters applied are detailed in Supplementary Material Table 2. Different numbers of bins (8, 16, 32, 64, 128, 256) and isotropic voxel sizes (1, 2, 3) were tested to assess the most robust quantisation/rebinning setting and confirm the number of bins with the largest set of robust features. To determine the most robust features against bin number and voxel size, approximately 10% of the total cohort was also re-segmented (*n* = 21). This created separate ROIs from which RFs were extracted and compared using interclass correlation coefficient (ICC).

**Table 2:** Mean training and validation scores for the best performing machine learning models along with hyperparameters and radiomic features selected

Machine learning model	Hyperparameters	Radiomic features selected	AUC mean training (SD)	AUC mean validation (SD)
Ridge Regression	C: 0.03, penalty: l2, solver: saga	Logarithm GLSZM Large Area Emphasis, wavelet-LLH GLCM ClusterProminence, wavelet-HLL GLCM MCC, wavelet-HLH_firstorder_Median, wavelet-HHH GLCM MCC	0.73 (0.02)	0.71 (0.10)
SVM	C: 3.6, degree: 6, ga2mma: 0.13, kernel: rbf	Exponential GLDM Small Dependence Low Grey Level Emphasis, wavelet-HLL first-order Mean, wavelet-HLL GLCM MCC, wavelet-HHH first-order Mean	0.88 (0.01)	0.70 (0.07)
KNN	algorithm: kd_tree, metric: manhattan, n_neighbors: 13, weights: uniform	Original firstorder 90^th^ percentile, wavelet-LLH GLCM Autocorrelation, wavelet-LHH first-order Maximum, wavelet-HHL first-order Skewness, wavelet-HHH GLCM MCC	0.78 (0.02)	0.70 (0.09)
Random forest	bootstrap: false, max_depth: 1, max_features: log2, min_samples_leaf: 5, min_samples_split: 32, n_estimators: 416	Original GLDM Large Dependence Low Grey Level Emphasis, exponential NGTDM strength, gradient first-order 10^th^ percentile', wavelet-LLH GLCM autocorrelation, wavelet-LHL GLCM correlation, wavelet-LHH first-order Entropy	0.79 (0.02)	0.69 (0.08)
Elastic net	C: 1e-06, l1 ratio: 1.25e-05, penalty: elastic net, solver: saga	Gradient first-order 10^th^ percentile, wavelet-LLH GLCM autocorrelation, wavelet-LHL GLCM Correlation, wavelet-LHH GLCM MCC, wavelet-HLL first-order entropy, wavelet-HLL GLCM MCC, wavelet-HLH first-order entropy, wavelet-HLH first-order Median, wavelet-HHH GLCM MCC	0.70 (0.03)	0.61 (0.10)
LASSO	C: 0.08, penalty: l1, solver: liblinear	wavelet-HLL GLCM MCC and wavelet-HHH GLCM MCC	0.69 (0.03)	0.62 (0.10)

AUC = area under the curve, SVM = support vector machine, KNN = k-nearest neighbour, LASSO = Least Absolute Shrinkage and Selection Operator, GLSZM = logarithm grey level size zone matrix, LAE = Large Area Emphasis, GLCM = Grey Level Co-occurrence Matrix, MCC = Maximal Correlation Coefficient (MCC), GLDM = Grey Level Dependence Matrix, NGTDM = Neighbouring Grey Tone Difference Matrix, SD = standard deviation

The Image Biomarkers Standardization Initiative (IBSI) was adhered to, which provides a comprehensive review of each step involved in radiomic analyses, including nomenclature of RFs and required calibration datasets [23]. Number of bins was favoured over the bin width given the arbitrary nature of MRI intensity units. The ComBat Harmonisation method (https://github.com/Jfortin1/ComBatHarmonization, accessed 09/02/2023) (v0.2.10) was applied to extracted RFs to account for variation in scanner models, acquisition protocols and reconstruction settings which RFs are affected by [30, 31].

### Feature selection

First, an unsupervised method of feature selection was applied to reduce the dataset using Pearson’s correlation coefficient. For each feature pair, correlations were assessed, a threshold of 0.8 was used to highlight highly correlated pairs and the feature in the pair with the largest average correlation to all other features was removed. Additional feature selection steps were performed using three different methods: a forward wrapper method (mlxtend 0.18.0); a univariate analysis method (scikit-learn v0.24.2); and a recursive feature extraction method (where applicable) (scikit-learn v0.24.2). A maximum of 5 features were chosen.

### Machine learning (ML) model construction and statistical analysis

The dataset was split into training and test sets stratified around MRI scanner vendor and ISUP, with an 80:20 split using scikit-learn (v0.24.2) (https://scikit-learn.org/stable/whats_new/v0.24.html, accessed 09/02/2023). Six predictive ML methods (as listed under ML model construction in Fig. 1) were implemented with the Python library scikit-learn (v23.0) in order to incorporate the selected RFs into a binary classifier for distinguishing patients grouped as hypoxia or normoxia [24]. Methods used included ridge regression, random forest (RFo), elastic net, k-nearest neighbour (KNN), support vector machine (SVM), and least absolute shrinkage and selection operator (LASSO) regression. These models were trained to build classification models based on whole prostate T2w RFs, respectively.

Training of ML models and tuning of hyperparameters was performed using a Bayes search cv (scikit-optimize v0.8.1), with fivefold cross-validation stratified around hypoxia status (normoxia or hypoxia) with 25 repeats. The area under the curve (AUC) of the receiver operating characteristic (ROC) curve was calculated with confidence intervals and the DeLong method was used to compare AUCs, to assess how accurately the radiomic and clinical-only models could classify a tumour’s hypoxia status [25]. RFs and hyperparameters with the highest mean validation AUC which was within 0.05 of the mean training AUC were selected. A 0.05 cut-off was chosen to try and minimise selection of an overfitted model. The model which had the highest mean validation AUC overall was tested once on the unseen test set. The overall evaluation of clinical variables between the training and testing groups to ensure balanced groups was compared using the independent t test (continuous variables) and Chi-square test (categorical variables). The statistically significant level was set at 0.05.

## Results

The demographics, pathology information and hypoxia status of the prostate tumours in the final study cohort, split by training and test cohort, are described in Table 1.

## Machine learning model building

**Fig. 2:**
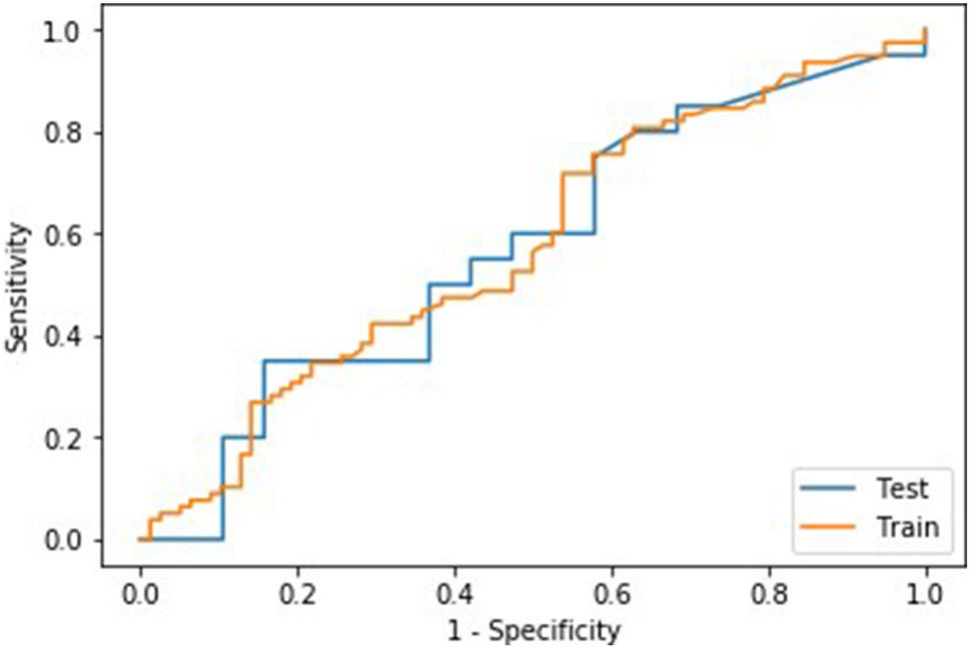
ROC curve of the best performing ridge regression hypoxia prediction model (test and training performance) using clinical features. Mean training AUC 0.61 (SD 0.02), mean training validation AUC 0.60 (SD 0.08). Mean test AUC 0.57 (95% CI 0.14)

The best performing model with clinical variables alone was a ridge regression model (Fig. 2) which included age and tumour stage variables. Mean training AUC was 0.61 (Standard Deviation (SD) 0.02), and mean training validation AUC was 0.60 (SD 0.08). Mean test AUC was 0.57 (95% confidence interval (CI) 0.14). The ML models with added RFs outperformed the clinical-only model. Mean training and validation AUCs for the best performing radiomics-based ML models along with hyperparameters and selected RFs are shown in Table 2.

**Fig. 3:**
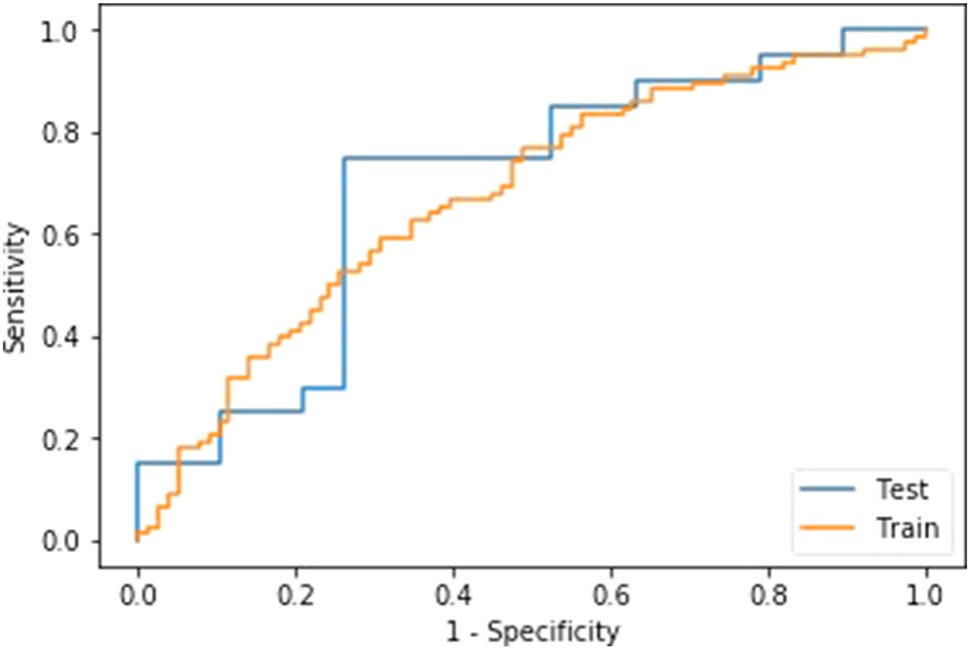
ROC curve of the best performing ridge regression hypoxia prediction model (test and training performance) using radiomic features. Mean training AUC 0.73 (SD 0.02). Mean training validation AUC 0.71 (SD 0.10). Mean test AUC 0.69 (95% CI 0.14).

The model within the highest mean validation AUC was a ridge regression model created using radiomic and clinical features. The best performing ML model is shown in Fig. 3 with a mean training AUC of 0.73 (SD 0.02), mean training validation AUC of 0.71 (SD 0.10) and mean test AUC of 0.69 (95% CI 0.14). The 5 selected RFs were logarithm grey level size zone matrix (GLSZM), Large Area Emphasis (LAE) and the following 3-dimensional wavelet features: LLH grey level co-occurrence matrix (GLCM) Cluster Prominence, HLL GLCM maximal correlation coefficient (MCC), HLH first-order Median, HHH GLCM MCC. No clinical features were selected despite integrating all clinical variables into the model.

**Fig. 4:**
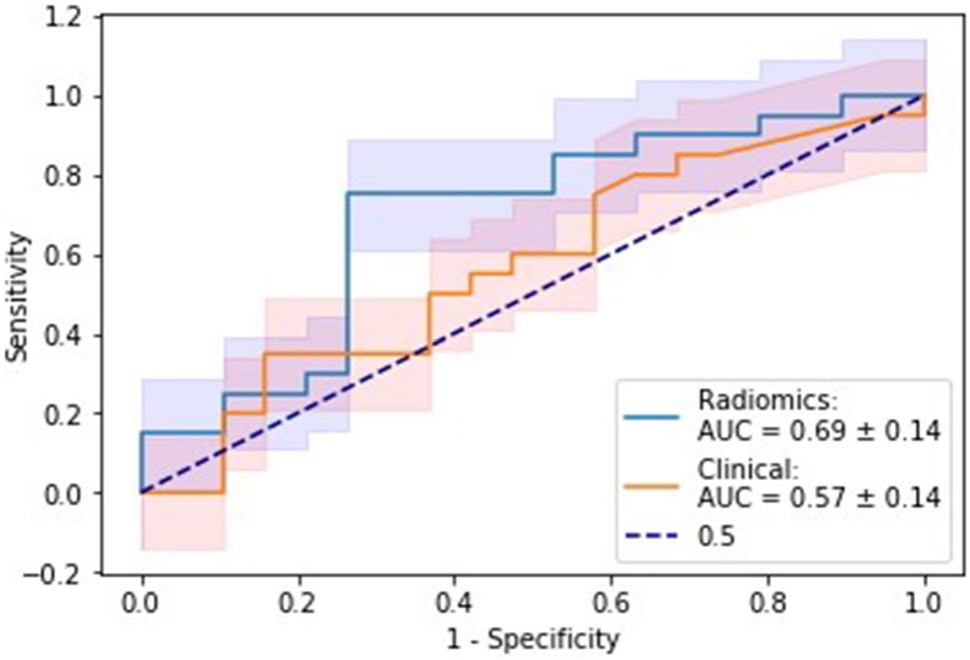
Mean ROC curves of the best performing radiomics and clinical-based ridge regression hypoxia prediction models with 95% confidence intervals highlighted

For the combined model with the highest AUC, performance metrics were: overall model accuracy 0.72, sensitivity 0.74, specificity 0.70, positive predictive value (PPV) 0.70, and negative predictive value (NPV) 0.74. The best performing clinical model had an overall accuracy of 0.56, sensitivity of 0.67, specificity of 0.53, PPV of 0.3, and NPV of 0.84. Confusion matrices are presented in Tables 3 and 4, respectively. The radiomics and clinical-only models showed no significant difference according to DeLong's testing (*p* = 0.35) (Fig. 4).

**Table 3:** Confusion matrix for clinical-based ridge regression hypoxia prediction model

Prediction	Negative	Positive
Predicted Negative	16	3
Predicted Positive	14	6

Positive = Hypoxia tumour status, Negative = Normoxia tumour status, Predicted Positive = predicted to be hypoxic, Predicted Negative = predicted to be normoxic

**Table 4:** Confusion matrix for radiomics-based ridge regression hypoxia prediction model

Prediction	Negative	Positive
Predicted Negative	14	5
Predicted Positive	6	14

Positive = Hypoxia tumour status, Negative = Normoxia tumour status, Predicted Positive = predicted to be hypoxic, Predicted Negative = predicted to be normoxic

## Discussion

The aim of this study was to develop a ML model based on RFs extracted from whole prostate gland T2w MRI for non-invasive prediction of tumour hypoxia identified in biopsies using a 32-gene signature [9]. The results showed that the integration of RFs from MRI helped improve the prediction of hypoxia in patients with prostate cancer, with the best ML model (ridge regression) having an AUC of 0.69 for the unseen internal test cohort compared to 0.57 for a model derived from only clinical variables. Although this change did not reach statistical significance, it still highlights the potential use of MRI to non-invasively assess hypoxia status in prostate cancer.

The benchmark for tumour hypoxia determination in this study was a hypoxia-associated gene expression (Ragnum) signature. This intrinsic molecular biomarker reflected the transcriptional profile associated with pimonidazole staining, an extrinsic marker of hypoxia, and was validated for prognostic significance in independent datasets [9]. Whole-mount prostate specimens were not available in the current cohort where all patients only received RT. By using the gene signature as the ground truth, we were able to provide a biological basis for the observed hypoxia-associated RFs selected by the ML models.

MRI-guided EBRT focal boosting to intra-prostatic lesions has been demonstrated to be safe and Level 1 evidence shows it improves biochemical control when compared to whole prostate EBRT [26]. Incorporating imaging radiogenomics into MRI-guided focal boosting of hypoxic tumours may further improve clinical outcomes given that hypoxic cells are three times more radioresistant than normoxic ones [8, 27]. Most prostate cancer patients undergo MRI routinely as part of diagnostic work-up, and T2w imaging is the most utilised sequence thereby potentially facilitating use of a T2w MRI hypoxia radiomics-based approach in the clinic. Despite the role of adaptive RT, there is no routine clinical use of any imaging methods to identify hypoxic regions. However, in the era of MRI-guided RT using MRI linear accelerators, there is potential to develop a radiomics-based hypoxia targeted radiotherapy methodological framework. This approach would also require robust harmonisation algorithms to account for the difference in field strength and MRI parameters on MR linear accelerators.

After prostate RT, the only validated biomarker for disease recurrence is prostate specific antigen (PSA) [28, 29]. The results of this preliminary study suggest that imaging biomarkers and RFs could offer further measurable longitudinal metrics that might be used to help guide post-treatment surveillance and survival predictions. This aligns with evidence from other tumours such as glioblastoma and renal cell cancer, which provide a biological basis for RFs [30, 31]. Beig et al. reported that RFs extracted from different regions of interest in 180 patients with glioblastoma such as enhancing tumour, necrotic tumour, and peri-tumoral regions were predictive of a hypoxia enrichment score based on 21 genes implicated in the hypoxia pathway of glioblastoma [32]. The top-eight features most associated with the hypoxia enrichment score included RFs which quantified structural heterogeneity and their imaging-based radiogenomic hypoxic signature was associated with survival [32]. Gao et al. derived a hypoxia-gene-related radiogenomic signature using RFs extracted from contrast-enhanced CT and found that this was significantly associated with prognosis in patients with renal cell cancer validating this in an independent cohort [31]. Such validation and insight into the biological characteristics of tumours is vital and needs to be replicated for prostate cancer pathways, which the current study attempts to address.

Previous research investigating the association between MRI and transcriptomic profiles in prostate cancer have suffered from low patient numbers, limiting transferability of results [33, 34]. To develop imaging biomarkers of prostate hypoxia, the availability of ‘ground truth’ data such as pathology or genomic profiling is critical to ensure the translational gap can be crossed allowing integration into routine care [35]. Leech et al. found that a radiomics model extracted from 88 T2w prostate MRIs (single axial slice used rather than a volume) could predict tumour hypoxia measured using pimonidazole stained prostatectomy specimens [36]. Their ML model used elastic net regularisation and repeated cross-validation to yield an AUC of 0.60 (SD 0.2) without a validation dataset but further demonstrates the feasibility of building a radiomics hypoxia model using T2w MRI. The RFs selected by their ML model were mainly shape-based features but also included the textural feature grey level size zone matrix (GLSZM) which was also one of the features selected by the best performing ML model in the current study. GLSZM quantifies grey level zones in an image, and GLSZM large area emphasis (LAE), one of the selected RFs in the best ML model in our study, measures the distribution of ‘large area size zones’, where a larger value indicates bigger zones with more coarse textures. Another RF selected was grey level co-occurrence matrix (GLCM) which reflects the spatial relationship among pixels and defines how frequently a combination of pixels is present. This potentially suggests a heterogeneous appearing prostate with more coarse textures associating with hypoxia.

There are a number of limitations to the study: Genomic profiling and MRIs were performed over several years and scanner technology and imaging protocols have evolved in the interim; imaging data used were all acquired on 1.5 T scanners and many did not have functional imaging sequences available as this was not routine at the time of the initial imaging acquisition. Similarly, the transcriptomic data were generated in small, old biopsies. As a result, only T2w imaging was used to develop radiomic models; whole prostate segmentations were used to extract RFs as not all cases had a visible tumour on anatomical imaging, and it was not possible to match the site of biopsy taken. Previous work has linked normal background prostate tissue with high-risk gene expression profiles highlighting the value for evaluating the whole gland [37]. Despite this study being a two-institution curated dataset, a further prospective validation cohort would allow for further testing of the reproducibility across different imaging equipment. Obtaining these radiogenomic datasets with matched clinical, imaging, pathology and genomic data remains challenging and requires further collaboration and formation of consortia with standardized methods for RF extraction. Establishing more multi-institutional collaborations with the potential to utilise novel transfer learning techniques will help expand our knowledge of genomics and imaging phenotypes in prostate cancer [38]. A general drawback to retrospective imaging research is the lack of imaging protocol standardisation, which differ significantly across institutions. In this study, ComBat harmonisation was used to minimize issues related to MRI data acquired on multiple scanners [39, 40]. Exploring the added role of functional MRI sequences in imaging hypoxia is vital to develop more sensitive diagnostic pathways. Hompland et al. investigated a novel MRI technique called intravoxel incoherent motion (IVIM) as an indirect measure of tumour hypoxia and validated this against the exogenous hypoxia marker pimonidazole [41]. Similarly, R2* maps from blood oxygen level-dependent (BOLD) MRI sequences have been found to have a high sensitivity for defining intra-prostatic tumour hypoxia [10]. A major barrier to clinical translation of these advanced imaging techniques is the poor spatial resolution that is required to fully sample the tumour microenvironment [42]. Utilising routinely acquired T2w MR data yields higher resolution prostate images allowing for better appreciation of structural differences. It is also less prone to artefacts compared to other functional sequences such as diffusion weighted imaging. Validating imaging biomarkers and RFs using gene expression signatures provides a biological basis but the external validation of any radiogenomic signature followed by further testing in the setting of a prospective randomised trial is essential to demonstrate value in clinical translation [43].

In conclusion, the current study suggests that whole prostate MRI-radiomics has the potential to non-invasively predict tumour hypoxia prior to radiotherapy. Further external validation of the hypoxia-associated radiomics model in predicting biochemical recurrence and clinical outcomes is required to determine the benefit of using the integrated information for patient stratification.

## Supplementary Information

Below is the link to the electronic supplementary material.

Supplementary file1 (DOCX 17 kb)

## Abbreviations

RT: Radiation therapy
EBRT: External beam radiation therapy
MRI: Magnetic resonance imaging
ML: Machine learning
RF: Radiomic feature
T2w: T2 weighted
Gy: Grey
HDR: High dose rate
BT: Brachytherapy
ISUP: International Society of Urological Pathology
PSA: Prostate-specific antigen
RNA: Ribonucleic acid
CLAIM: Checklist for Artificial Intelligence in Medical Imaging
NIfTI: Neuroimaging Informatics Technology Initiative
ROI: Regions of interest
ICC: Interclass correlation coefficient
IBSI: Image Biomarkers Standardisation Initiative
RFo: Random forest
SVM: Support vector machine
LASSO: Least absolute shrinkage and selection operator
AUC: Area under the curve
ROC: Receiver operating characteristic
CI: Confidence interval
GLSZM: Grey level size zone matrix
LAE: Large Area Emphasis
GLCM: Grey level co-occurrence matrix
MCC: Maximal correlation coefficient
PPV: Positive predictive value
NPV: Negative predictive value
LLH, HLL, HLH, HHH: 3D wavelet radiomic features
IVIM: Intravoxel incoherent motion
BOLD: Blood oxygen level dependent
