# Supplementary material for: Prediction of prostate tumour hypoxia using pre-treatment MRI-derived radiomics: preliminary findings
Source: Radiol Med. 2023 May 17;128(6):765–74. doi: 10.1007/s11547-023-01644-3 (PMC10264289; doi:10.1007/s11547-023-01644-3)
Supplement: Supplementary file 1 — Supplementary file1 (DOCX 17 kb) [file 11547_2023_1644_MOESM1_ESM.docx]

Table 1: MRI acquisition parameters of the 4 different 1.5T MRI Scanners used to acquire prostate MRI data.

| **1.5T MRI Scanner** | **Repetition time/ echo time (ms)** | **Flip Angle (°)** | **Field of view (mm)** | **Matrix size (mm)** | **Slice thickness (mm)** |
| --- | --- | --- | --- | --- | --- |
| GE Signa | 5020/122 | 90 | 250 | 384 x 224 | 3 |
| Philips Achieva | 3500/ 90 | 90 | 220 | 256 x 192 | 3 |
| Siemens Aera | 3540/ 99 | 160 | 220 | 320 × 256 | 3 |
| Siemens Sola | 4500/ 88 | 150 | 200 | 320 × 256 | 3 |

Table 2: Radiomic feature classes that were extracted from the T2-weighted MRI whole prostate gland segmentations.

All feature classes listed below, with the exception of the shape-based features can be calculated on the original image and/or a derived image, such as applying one of several filters which is how the wavelet features were generated.

Individual feature description can be found at: https://pyradiomics.readthedocs.io/en/latest/features.html

| **First-order** |
| --- |
| 10^th^ Percentile |
| 90^th^ Percentile |
| Energy |
| Entropy |
| Interquartile Range |
| Kurtosis |
| Maximum |
| Mean Absolute Deviation |
| Mean |
| Median |
| Minimum |
| Range |
| Robust Mean Absolute Deviation |
| Root Mean Squared |
| Skewness |
| Total Energy |
| Uniformity |
| Variance |
| **Shape-based (3D)** |
| Mesh Volume |
| Voxel Volume |
| Surface Area |
| Surface Area to Volume ratio |
| Sphericity |
| Maximum 3D diameter |
| Maximum 2D diameter (Slice) |
| Maximum 2D diameter (Column) |
| Maximum 2D diameter (Row) |
| Major Axis Length |
| Minor Axis Length |
| Least Axis Length |
| Elongation |
| Flatness |
| **Gray Level Co-occurrence Matrix (GLCM)** |
| Autocorrelation |
| Cluster Prominence |
| Cluster Shade |
| Cluster Tendency |
| Contrast |
| Correlation |
| Difference Average |
| Difference Entropy |
| Difference Variance |
| ID: Inverse Difference |
| IDM: Inverse Difference Moment |
| IDMN: Inverse Difference Moment Normalized |
| IDN: Inverse Difference Normalized |
| IMC1: Informational Measure of Correlation 1 |
| IMC2: Informational Measure of Correlation 1 |
| Inverse Variance |
| Joint Average |
| Joint Energy |
| Joint Entropy |
| MCC: Maximal Correlation Coefficient |
| Maximum Probability |
| Sum Average |
| Sum Entropy |
| Sum Squares |
| **Gray Level Dependence Matrix (GLDM)** |
| Dependence Entropy |
| Dependence NonUniformity |
| Dependence NonUniformity Normalized |
| Dependence Variance |
| Gray Level NonUniformity |
| Gray Level Variance |
| High Gray Level Emphasis |
| Large Dependence Emphasis |
| Large Dependence High Gray Level Emphasis |
| Large Dependence Low Gray Level Emphasis |
| Low Gray Level Emphasis |
| Small Dependence Emphasis |
| Small Dependence High Gray Level Emphasis |
| Small Dependence Low Gray Level Emphasis |
| **Gray Level Run Length Matrix (GLRLM)** |
| Gray Level NonUniformity |
| Gray Level NonUniformity Normalized |
| Gray Level Variance |
| High Gray Level Run Emphasis |
| Long Run Emphasis |
| Long Run High Gray Level Emphasis |
| Long Run Low Gray Level Emphasis |
| Low Grey Level Run Emphasis |
| Run Entropy |
| Run Length NonUniformity |
| Run Length NonUniformity Normalized |
| Run Percentage |
| Run Variance |
| Short Run Emphasis |
| Short Run High Gray Level Emphasis |
| Short Run Low Gray Level Emphasis |
| **Gray Leven Size Zone Matrix (GLSZM)** |
| Gray Level NonUniformity |
| Gray Level NonUniformity Normalized |
| Gray Level Variance |
| High Gray Level Zone Emphasis |
| Large Area Emphasis |
| Large Area High Gray Level Emphasis |
| Large Area Low Gray Level Emphasis |
| Low Gray Level Zone Emphasis |
| Size Zone NonUniformity |
| Size Zone NonUniformity Normalized |
| Small Area Emphasis |
| Small Area High Gray Level Emphasis |
| Small Area Low Gray Level Emphasis |
| Zone Entropy |
| Zone Percentage |
| Zone Variance |
| **Neighboring Gray-Tone Difference Matrix (NGTDM)** |
| Busyness |
| Coarseness |
| Complexity |
| Contrast |
| Strength |
